# TLR3 contributes to persistent autophagy and heart failure in mice after myocardial infarction

**DOI:** 10.1111/jcmm.13328

**Published:** 2017-09-25

**Authors:** Ting Gao, Shao‐Ping Zhang, Jian‐Fei Wang, Li Liu, Yin Wang, Zhi‐Yong Cao, Qi‐Kuan Hu, Wen‐Jun Yuan, Li Lin

**Affiliations:** ^1^ Department of Physiology and Key Lab of Ministry of Education in Fertility Preservation and Maintenance Ningxia Medical University Yinchuan China; ^2^ Department of Physiology Shanghai Jiao Tong University School of Medicine Shanghai China; ^3^ Department of Ultrasound Shanghai Punan Hospital Shanghai China; ^4^ Ultrasound Department of Shanghai Pulmonary Hospital Tongji University Shanghai China; ^5^ Department of General Internal Medicine Branch of 411 Hospital of People's Liberation Army Shanghai China; ^6^ Department of Physiology Second Military Medical University Shanghai China

**Keywords:** toll‐like receptor, autophagy, myocardial infarction, heart failure

## Abstract

Toll‐like receptors (TLRs) are essential immunoreceptors involved in host defence against invading microbes. Recent studies indicate that certain TLRs activate immunological autophagy to eliminate microbes. It remains unknown whether TLRs regulate autophagy to play a role in the heart. This study examined this question. The activation of TLR3 in cultured cardiomyocytes was observed to increase protein levels of autophagic components, including LC3‐II, a specific marker for autophagy induction, and p62/SQSTM1, an autophagy receptor normally degraded in the final step of autophagy. The results of transfection with a tandem mRFP‐GFP‐LC3 adenovirus and use of an autophagic flux inhibitor chloroquine both suggested that TLR3 in cardiomyocytes promotes autophagy induction without affecting autophagic flux. Gene‐knockdown experiments showed that the TRIF‐dependent pathway mediated the autophagic effect of TLR3. In the mouse model of chronic myocardial infarction, persistent autophagy was observed, concomitant with up‐regulated TLR3 expression and increased TLR3‐Trif signalling. Germline knockout (KO) of TLR3 inhibited autophagy, reduced infarct size, attenuated heart failure and improved survival. These protective effects were abolished by *in vivo* administration of an autophagy inducer rapamycin. Similar to the results obtained in cultured cardiomyocytes, TLR3‐KO did not prevent autophagic flux in mouse heart. Additionally, this study failed to detect the involvement of inflammation in TLR3‐KO‐derived protection, as wild‐type and TLR3‐KO hearts were comparable in inflammatory activity. It is concluded that up‐regulated TLR3 expression and signalling contributes to persistent autophagy following MI, which promotes heart failure and lethality.

## Introduction

The family of toll‐like receptors (TLRs) serves as a critical component of the immune system 1. Ten functional TLRs have been identified in humans. Among them, TLR 1/2/4/5/6/10 are expressed on cell surface, whereas TLR3/7/8/9 are localized in intracellular vesicles such as endoplasmic reticulum and endosomes 2. Through recognizing and binding with invading microbes and endogenous danger molecules released from stressed cells, known as pathogen‐associated molecular patterns (PAMPs) and damage‐associated molecular patterns (DAMPs) respectively, TLRs can trigger efficient immune responses against microbial pathogens and cellular injury 3.

All TLRs are type I transmembrane proteins, composed of a leucine‐rich repeat (LRR) ectodomain associated with PAMP recognition, a single transmembrane domain, and a cytoplasmic Toll/IL‐1 receptor (TIR) domain involved in signal transduction. TLR signalling can be largely divided into two pathways, dependent on myeloid differentiation factor 88 (MyD88) and TIR‐domain‐containing adaptor inducing interferon‐β (Trif), respectively. The MyD88‐dependent pathway is universally used by all TLRs except TLR3, which activates nuclear factor‐κB (NF‐κB), a major inflammatory transcription factor, and induces inflammatory cytokine production. The Trif‐dependent pathway can be activated by TLR3 and TLR4, which activates interferon regulatory factor (IRF) 3 and NF‐κB, and consequently induces the production of type I interferon and inflammatory cytokines 2.

In addition to the MyD88‐ and Trif‐dependent immune pathways, autophagy has emerged as an effector mechanism downstream of TLRs. Autophagy is a conserved lysosomal degradation pathway responsible for eliminating protein aggregates and damaged organelles. In mammalian cells, autophagy comprises at least three distinct pathways, macroautophagy, microautophagy and chaperone‐mediated autophagy. The term ‘autophagy’ generally refers to macroautophagy unless otherwise specified. We hereafter refer to macroautophagy as autophagy. Autophagy occurs at basal levels and can be up‐regulated by multiple stresses such as nutrient starvation and ischaemia. There is much controversy over the role of autophagy in cell survival and cell death under stresses 4.

Several TLR subtypes have been described to induce autophagy in immune cells such as macrophages and dendritic cells, which facilitates elimination of invading pathogens 5, 6, 7, 8. However, little is known about the relation of TLRs to autophagy in other cell types. It is notable that non‐immune cells, such as cardiomyocytes, express multiple TLR subtypes 9. Although autophagy serves as an essential component for physiological and pathological processes in the heart 10, it remains unknown whether TLRs regulate autophagy in cardiomyocytes. Our interest was directed to TLR3, an intracellular subtype predominantly expressed by cardiomyocytes 11. In a preliminary study, we observed that the activation of TLR3 enhanced autophagic activity in cultured cardiomyocytes. This study was designed to examine the effect of TLR3 on cardiac autophagy under conditions of ischaemic stress.

## Materials and methods

### Primary culture of neonatal rat ventricular myocytes (NRVMs)

The neonatal Sprague‐Dawley rats were sacrificed by decapitation and used for the preparation of the primary culture of ventricular myocytes, as we described previously 12. Briefly, the ventricles were removed, rinsed, minced and digested with 0.2% trypsin (Gbico™ Cat. 27250‐018: Thermo Fisher Scientific Inc., Shanghai, China) in Ca^2+^‐ and Mg^2+^‐free Hanks solution for repeated short time periods. Collected cells were filtered through a nylon mesh, centrifuged, resuspended in Dulbecco's modified Eagle's medium supplemented with foetal bovine serum, pre‐plated, then cultured in a humidified atmosphere of 5% CO_2_ at 37°C.

In the gene‐knockdown experiments, NRVMs were transfected with small interference RNA (siRNA) duplexes. The siRNA against TLR3 followed the literature 13, and the other siRNAs were designed and synthesized by Shanghai GenePharma Co., Ltd, Shanghai, China. Pilot experiments were preformed to test their efficacy. All siRNAs were administered at the final concentration of 50 nM, with the aid of Lipofectamine^®^ RNAiMAX transfection reagent (Thermo Fisher Scientific Inc., Shanghai, China), following the manufacturer's instructions. The sequences of siRNAs are as follows: negative control, 5′‐TTCTCCGAACGTGTCACGT‐3′; TLR3, 5′‐GGTGTCTTCCACAAACCAA‐3′; MyD88, 5′‐GCCAGAAATACATACGCAA‐3′; Trif, 5′‐GCCTGGGATCGGTGTAGTT‐3′.

### Use of tandem mRFP‐GFP‐LC3 to assess autophagic flux

To assess autophagic flux in response to TLR3 activation, a tandem mRFP‐GFP‐LC3 adenovirus (Hanheng Biotechnology Co Ltd., Shanghai, China) was transfected into cultured NRVMs for 24 hrs at a MOI of 50. The tandem mRFP‐GFP‐LC3 protein shows both red (mRFP) and green (GFP) fluorescence in neutral pH. It can form yellow (red+green) puncta that represent autophagosome formation. When an autophagosome fuses with a lysosome, the GFP moiety is degraded as it is pH‐labile, but mRFP–LC3 maintains the puncta, which then tracks the autolysosomes 14. The relative ratio of red‐only *versus* yellow puncta is an index of autophagic flux.

### Mice model of myocardial infarction

TLR3^‐/‐^ mice in the background of C57BL/6 were purchased from the Jackson Laboratory (Stock No: 009675), and wild‐type (WT) C57BL/6 mice were purchased from SIPPR‐BK Laboratory Animal Co. Ltd., Shanghai, China. A mice model of myocardial infarction (MI) was prepared as described previously 15. Mice (8–10 weeks of age) were anaesthetized with 2% isoflurane mixed with oxygen (1.5 l/min.). The adequacy of anaesthesia was checked by the lack of corneal reflex and withdrawal reflex to toe pinch. The chest was depilated, a skin cut was made on the left side and a small hole was made under the fourth rib using a mosquito clamp. The clamp was slightly open to allow the heart to ‘pop out’ through the hole. Then, the left anterior descending coronary artery (LAD) was sutured and ligated with a 6/0 braided silk suture, at the site approximately 2 mm from its origin. MI was confirmed by visual cyanosis of the heart. After ligation, the heart was immediately placed back into the intrathoracic space, and the chest was closed. Sham mice received the same procedure except that LAD was not ligated.

At the end of the 4‐week observation period and after echocardiography, the mice were euthanized by placing into a chamber filled with vapour of isoflurane until respiration ceased, and heart tissue was then collected for examination. In a subgroup of mice, the autophagy inducer rapamycin (2 mg/kg/day) or autophagic flux inhibitor chloroquine (50 mg/kg/day) was daily intraperitoneally injected, starting from 24 hrs after surgery and lasting through the observation period of 2 weeks.

All animal procedures were approved by the Animal Experiment Committee of Ningxia Medical University, in accordance with the Guide for the Care and Use of Laboratory Animals published by the US National Institutes of Health (8th Edition, 2011).

### Haematoxylin and eosin (HE) staining and Masson's trichrome staining

HE staining and Masson's trichrome staining were performed for histopathological observation. After euthanasia, the heart was isolated, perfused with normal saline followed by 4% paraformaldehyde for fixation, dehydrated with ethanol, coronally sectioned into halves along the long axis, embedded in paraffin blocks, consecutively sectioned into 5‐μm‐thick slices, and then stained with commercial reagents for HE or Masson's trichrome. In HE staining, nuclei are stained blue‐purple by haematoxylin, whereas cytoplasm and extracellular matrix have varying degrees of pink staining. In Masson's staining, muscle fibres are stained purple‐red, while collagen fibres are stained green‐blue.

### Infarct size measurement

The infarct size was determined with a length‐based approach described previously 16. Coronal slices of the heart were prepared and stained with Masson's trichrome as described above. Using Masson's images of the whole pieces of coronal slices, myocardial midline was drawn at the centre between the epicardial and endocardial surfaces of left ventricle (LV), and the total length of LV midline was recorded as midline circumference. Meanwhile, midline infarct length was taken as the midline of the length of infarct that included >50% of the whole thickness of the myocardial wall. Infarct size was calculated as the percentage of midline infarct length relative to LV circumference.

### Echocardiographic examination

Transthoracic echocardiography was performed at the end of the observation period to determine heart function, using an ultrasonic apparatus (Voluson E8; GE Healthcare, General Electric Co., Farmingdale, NY, USA. 15‐MHz probe) 17. Under the anaesthesia of isoflurane, the short‐axis view of mouse heart was acquired at the papillary muscle level through two‐dimensional mode, and consecutive M‐mode images in the short‐axis view were recorded. Left ventricular end‐diastolic diameter (LVEDD) and end‐systolic diameter (LVESD) were measured from M‐mode tracings, and fractional shortening (FS) was calculated as (LVEDD‐LVESD)/LVEDD × 100%.

### Real‐time reverse transcription‐polymerase chain reaction (RT‐PCR)

The mRNA levels of target genes in mouse heart were measured using the method of real‐time RT‐PCR 18. Briefly, total RNA was extracted with Trizol (Invitrogen, Shanghai, China) following the manufacturer's instructions, reversely transcribed into cDNA using oligo‐dT primers and then performed with real‐time quantitative PCR using SYBR Green (Qiagen, Shanghai, China) as the reporter dye. All cDNA samples were analysed in duplicate. The relative level of target mRNA was calculated by the method of 2^−∆∆Ct^, and 18S ribosomal RNA was used as the loading control. The primer sets for real‐time PCR are as follows: TLR3, 5′‐CCTATGGATTCTTCTGGTGTC‐3′ and 5′‐TCTTCTGAGTTGGTTGTGAGT‐3′; TNFα, 5′‐GCCTCCCTCTCATCAGTTCT‐3′ and 5′‐ACTTGGTGGTTTGCTACGAC‐3′; IL‐6, 5′‐CCAATGCTCTCCTAACAGAT‐3′ and 5′‐TGTCCACAAACTGATATGCT‐3′; 18S RNA, 5′‐CGTCTGCCCTATCAACTTTC‐3′ and 5′‐GGATGTGGTAGCCGTTTCT‐3′.

### Western blot and co‐immunoprecipitation (co‐IP) analysis

Western blot and co‐IP experiments were performed as we described previously 18. RIPA buffer containing protease inhibitors was used to extract total proteins from heart tissue and cultured cardiomyocytes, and protein concentration was determined using a bicinchoninic acid kit. For Western blot, 20 μg of total proteins was electrophoresed on a 10% SDS‐PAGE gel and transferred onto a nitrocellulose membrane. The membrane was then blocked with 5% non‐fat dried milk, probed with specific primary antibodies (1:500–1000) followed by peroxidase‐conjugated secondary antibodies (1:1000) and visualized using chemiluminescence reagents. Western blotting signal was quantified by densitometry. The primary antibodies against TLR3 (Cat. NB100‐56571), MyD88 (Cat. NBP1‐19785) and Trif (Cat. NB120‐13810) were purchased from Novus Biologicals, LLC, Littleton, CO, USA; the anti‐LC3 antibody (Cat. AL221) was from Beyotime Institute of Biotechnology, Jiangsu, China; the anti‐beclin‐1 antibody (Cat. 11306‐1‐AP) was from Proteintech Group, Inc., Rosemont, IL, USA; and the anti‐p62 antibody (Cat. 5114) was from Cell Signalling Technology, Inc., Danvers, MA, USA.

Co‐IP experiments were performed to examine the interaction between TLR3 and MyD88/Trif, and between TLR3 and LC3/beclin‐1. For co‐IP, the tissue lysates (300 μg of total proteins) were pre‐incubated with 2 μg IgG of the same isotype as the primary antibody to block non‐specific combination, followed by incubation with 6 μg anti‐TLR3 antibody (Cat. sc‐8691, Santa Cruz Biotechnology, Inc., Santa Cruz, CA, USA) overnight at 4°C. Protein G‐agarose beads were then added to precipitate the antibody complexes. The precipitates were then subjected to regular Western blot analysis as described above.

### Immunohistochemical staining

Immunohistochemical staining was carried out to examine the expression of TLR3 in the heart. Paraffin‐embedded heart slices were used for the staining. Briefly, the slices were dewaxed, microwaved to retrieve antigen, blocked with 5% BSA, incubated overnight at 4°C with an isotype IgG control antibody, or with a primary antibody against TLR3 (diluted 1:50, Cat. sc‐8691, Santa Cruz Biotechnology, Inc.), and then hybridized with horseradish peroxidase (HRP)‐labelled second antibodies followed by 3,3′‐diaminobenzidine (DAB) chromogenic reaction. The brown DAB deposits were observed under a microscope.

### Transmission electron microscopy

Transmission electron microscopy was used to observe autophagic vacuoles. Heart tissue was quickly cut into1 mm cubes, fixed with 2.5% glutaraldehyde overnight at 4°C, immersed in 1% osmium tetroxide for 2 hrs, dehydrated in graded ethanol, embedded in epoxy resin and incised into ultrathin sections (60–70 nm). The sections were then double stained with uranylacetate and lead citrate and examined under a Hitachi H‐7650 transmission electron microscope (JEOL, Peabody, MA, USA).

### Statistics and data analysis

All the data are expressed as means ± S.D. Differences between multiple groups were analysed by the one‐way analysis of variance (anova) followed by Fisher's least significant difference (LSD) test, using SAS 9.0 statistical software (SAS Institute Inc., Cary, NC, USA). Differences between two groups were analysed by unpaired *t*‐test. A *P *<* *0.05 (two‐tailed) was considered statistically significant.

## Results

### TLR3 agonist induced autophagy in cultured cardiomyocytes through a TRIF‐dependent pathway

**Figure 1:**
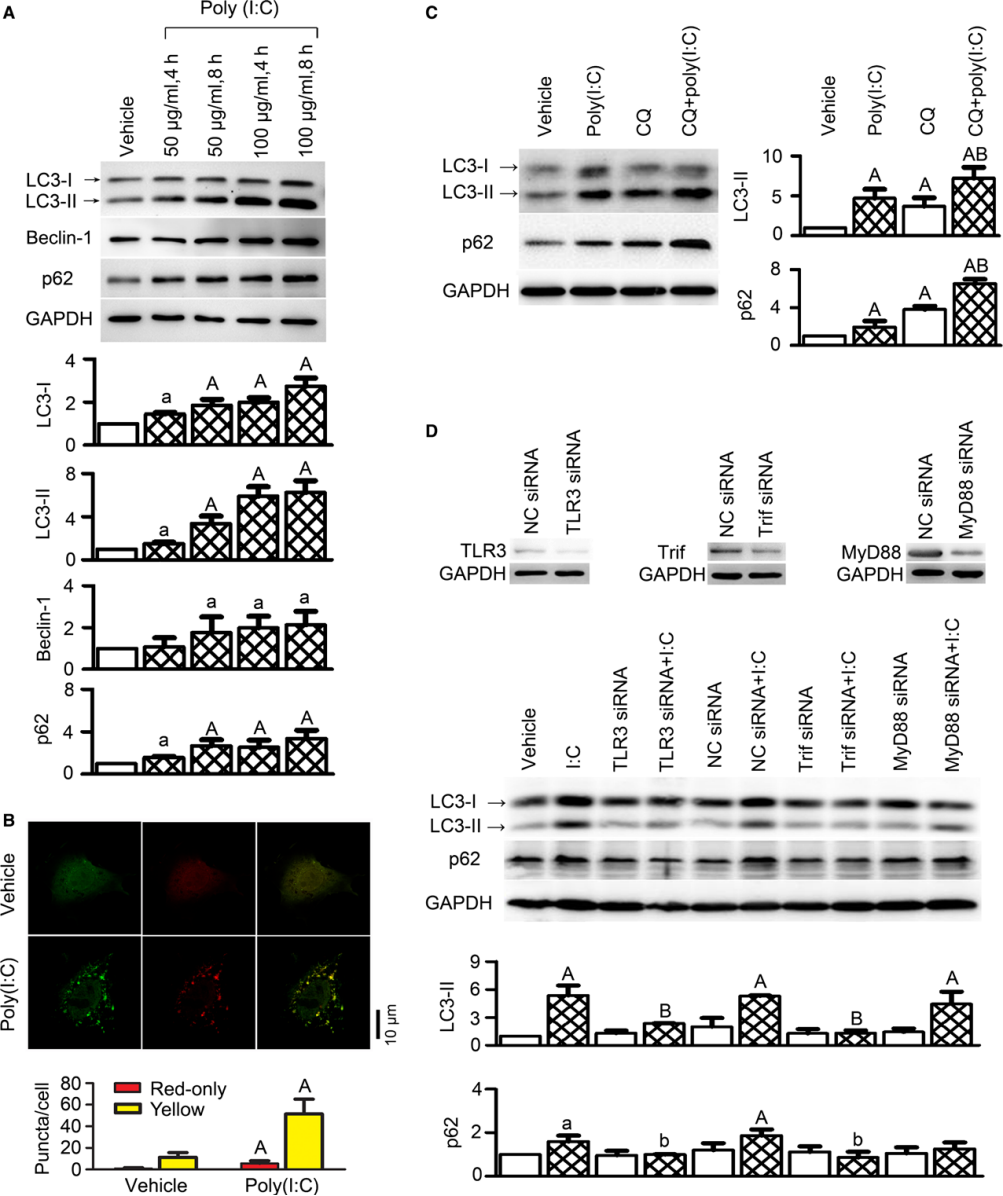
A TLR3 agonist polyinosinic‐polycytidylic acid (poly(I:C)) induced autophagy in cultured cardiomyocytes through a TRIF‐dependent pathway. (**A**) Poly(I:C) increased autophagy markers in cultured H9c2 rat ventricular cells. (**B**) Poly(I:C) stimulated autophagosome formation but did not affect autophagic flux. Primary cultured neonatal rat ventricular myocytes (NRVMs) were transfected with a tandem mRFP‐GFP‐LC3 adenovirus for 24 hrs, followed by treatment with poly(I:C) (100 μg/ml, 4 hrs). Autophagosomes and autolysosomes were, respectively, visualized as yellow‐ and red‐only punctas under a confocal microscope. (**C**) An autophagic flux inhibitor chloroquine (CQ) induced accumulations of LC3‐II and p62/SQSTM1 proteins in H9c2 myocytes receiving poly(I:C) (100 μg/ml, 4 hrs). CQ was applied at 10 μM, immediately prior to poly(I:C). (**D**) Effects of indicated siRNA on poly(I:C)‐induced changes in autophagy markers in NRVMs. All the siRNAs were transfected at 50 nM for 48 hrs, and poly(I:C) was added at 100 μg/ml for 4 hrs before cell harvest. Negative control (NC) siRNA served as control. RNAiMAX transfection reagent was used in all the siRNA experiments. The upper panel shows the knockdown effects of siRNAs, and the lower panel shows representative Western blot images (presented from four independent experiments) and densitometry quantitative data (normalized into ‘fold of vehicle group’). All quantitative data are expressed as means ± S.D. ^a^ *P* < 0.05, ^A^ *P* < 0.01 *versus* vehicle; ^b^ *P* < 0.05, ^B^ *P* < 0.01 *versus* poly(I:C).

To examine the effect of TLR3 on cardiac autophagy, we used a synthetic ligand for TLR3 (polyinosinic‐polycytidylic acid, poly (I:C)) to treat cultured cardiac myocytes, and examined autophagy markers including microtubule‐associated protein 1 light chain 3 (LC3), beclin‐1 and p62/SQSTM1. LC3 is a specific marker for autophagy initiation. It exists as an 18‐kD cytosolic LC3‐I form in resting cells and is lipidated upon autophagy induction to produce autophagosome‐associated LC3‐II, which migrates in SDS‐PAGE as a 16‐kDa protein 19, 20. The quantification of LC3‐II protein level normalized to a loading control is essential for autophagy measurements 21. P62/SQSTM1 is an ubiquitin‐binding adaptor protein that is removed in the final digestion step of autophagy. It acts as an autophagy receptor, binding directly to LC3 to facilitate degradation of ubiquitinated protein aggregates 22, 23. Using the method of Western blot, we observed that treatment with poly(I:C) in myocytes induced a large increase in LC3‐II proteins, accompanied by relatively smaller increases in LC3‐I, beclin‐1 and p62/SQSTM1 proteins (Fig. 1A). These results suggested that poly(I:C) up‐regulated autophagic activity in cardiac myocytes.

As the increase in LC3‐II may result from either induction of autophagy or reduction in autophagic flux (the rate of transit of autophagosome cargo through lysosomal degradation), we subsequently examined whether TLR3 affects autophagic flux. A tandem mRFP‐GFP‐LC3 adenovirus was transfected into NRVMs for 24 hrs, followed by treatment with poly(I:C) (100 μg/ml, 4 hrs). Significant increases in the numbers of autophagosomes (yellow puncta) and autolysosomes (red‐only puncta) were observed after poly(I:C) treatment (Fig. 1B). However, the relative ratio of red‐only to yellow puncta was unchanged, suggesting no change in autophagic flux. Furthermore, we determined LC3‐II and p62/SQSTM1 protein levels in the absence and presence of chloroquine (CQ), an autophagic flux inhibitor that prevents autophagosome‐lysosome fusion and lysosomal degradation 24. As shown in Fig. 1C, CQ led to similar accumulations of LC3‐II and p62/SQSTM1 proteins, despite the presence or absence of poly(I:C), suggesting that TLR3 activation did not affect autophagic flux. Taken together, it is suggested that autophagy induction was enhanced by TLR3 activation in cardiac myocytes, whereas autophagic flux remained intact.

To further dissect the signalling mechanism of TLR3‐mediated autophagy, MyD88 and Trif were individually knocked‐down by siRNAs in NRVMs. The results showed that Trif siRNA attenuated increases of LC3‐II and p62/SQSTM1 caused by poly(I:C) (Fig. 1D). In contrast, neither negative control (NC) nor MyD88 siRNA showed significant effects. The above data suggest that TLR3 stimulation promotes autophagy induction in cardiac myocytes through a TRIF‐dependent pathway.

### TLR3‐knockout inhibited MI‐induced persistent autophagy in mouse heart

To investigate the potential autophagic effect of cardiomyocyte TLR3 *in vivo*, we employed the model of MI, which has been described to drive autophagy in the heart 10, 25. The function of TLR3 in MI‐induced autophagy was examined here using TLR3‐knockout (TLR3‐KO) mice.

**Figure 2:**
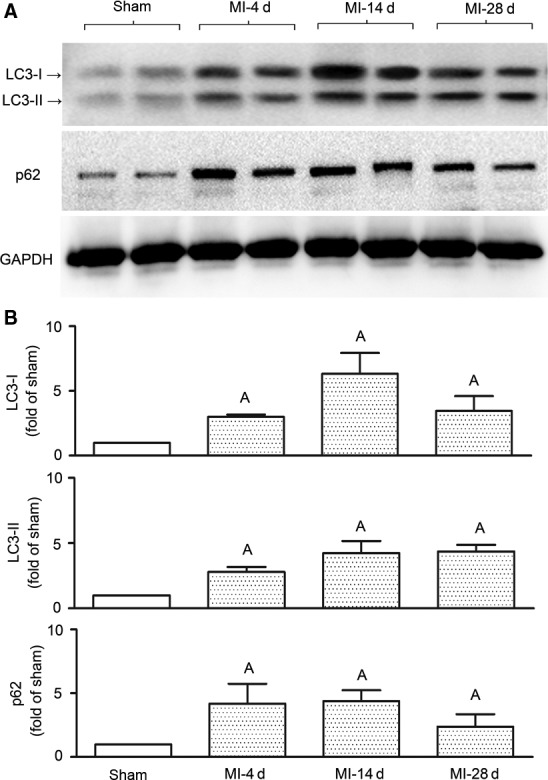
Autophagy is persistently induced in myocardium after MI. Autophagy markers in infarct myocardium were determined by Western blotting on days 4, 14 and 28 after the surgery of myocardial infarction (MI). (**A**) Representative Western blot images. (**B**) Quantitative analyses. *n *=* *4/group. Data are means ± S.D. ^A^ *P* < 0.01 *versus* sham.

Firstly, to examine autophagy, we monitored the dynamic changes of LC3 and p62/SQSTM1 in infarct myocardium after MI. The results (Fig. 2) showed that LC3‐II accumulated in a time‐dependent manner. A mild increase of LC3‐II was detected on day 4 after MI, which was dramatically enhanced by 2 weeks and remained significantly up‐regulated at 4 weeks. The level of p62/SQSTM1 increased in a similar pattern to LC3‐II. Theoretically, p62/SQSTM1 increase can result from enhanced autophagy induction and/or decelerated autophagic flux. An autophagic stimulus typically induces an early increase in p62/SQSTM1, followed by clearance of p62/SQSTM1 associated with autophagosome cargo. Consequently, when flux is intact, p62/SQSTM1 barely changes at equilibrium up‐regulated autophagy, whereas when flux is impaired, p62/SQSTM1 will rise dramatically 21. Here we detected increases of p62/SQSTM1 after MI, which was likely attributable to persistent activation of autophagy. A proof is that while p62/SQSTM1 dropped by day 28, compared to day 4 and day 14, LC3‐II consistently remained at a high level (Fig. 2). Furthermore, the autophagic flux inhibitor CQ increased LC3‐II and p62/SQSTM1 in infarcted hearts of wild‐type mice as shown afterwards (Fig. 6A). It is suggested that autophagy was persistently induced in the heart after MI, whereas autophagic flux was barely affected.

**Figure 3:**
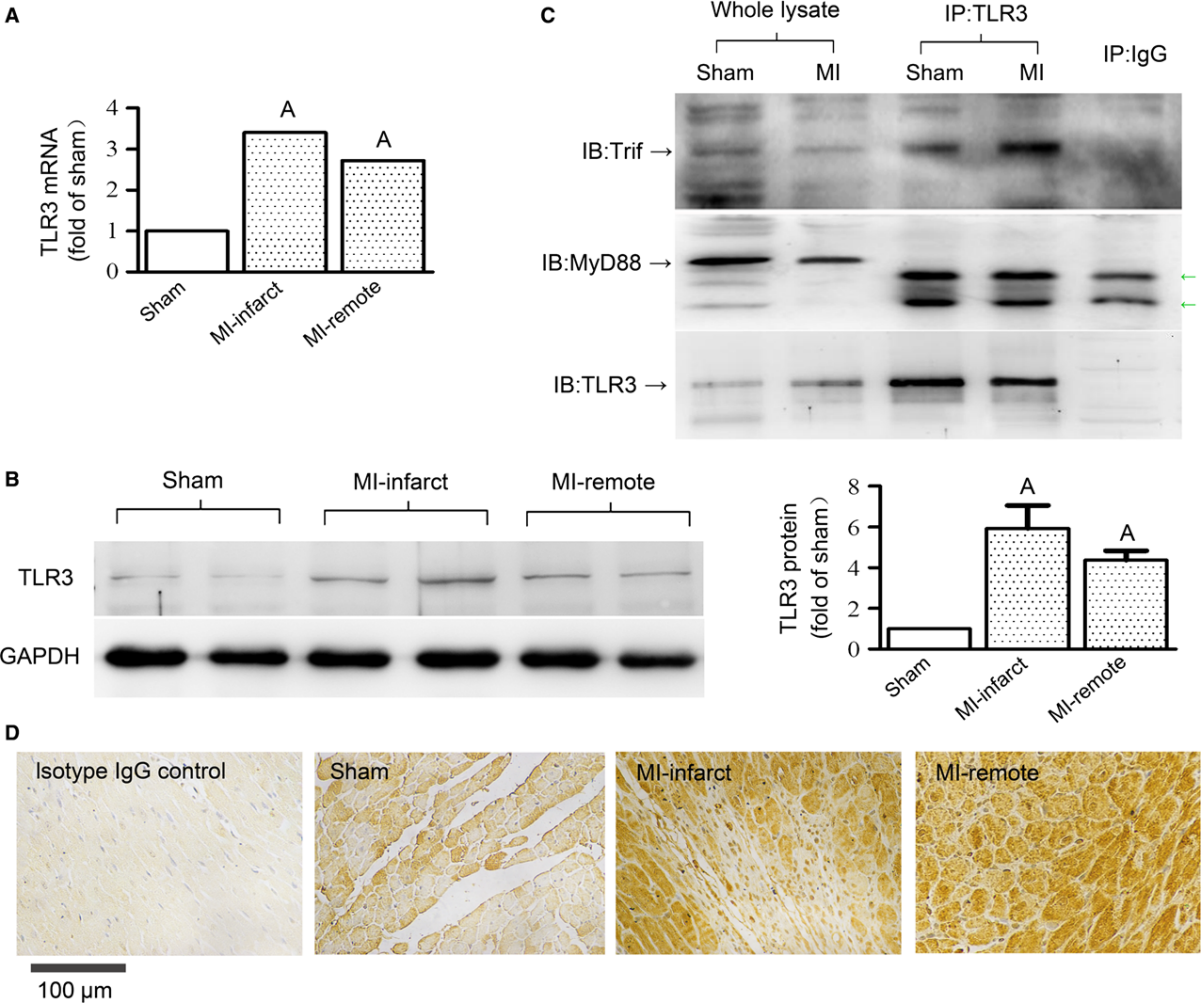
Myocardial infarction (MI) increased TLR3 expression and its physical association with Trif in mouse myocardium. Heart samples were taken from infarct area at 4 weeks after MI. (**A**) and (**B**) show mRNA and protein levels of TLR3 in sham and MI hearts. *n *=* *4 mice/group. Data are means ± S.D. ^A^ *P* < 0.01 *versus* sham. (**C**) Representative immunohistochemistry images of heart sections stained for TLR3 (brown colour). An isotype IgG control was performed to verify the specificity of TLR3 reactivity. (**D**) Lysates of heart tissue were immunoprecipitated with anti‐TLR3 antibodies (IP: TLR3), followed by SDS–PAGE and immunoblotting (IB) with indicated antibodies. IP with isotype IgG (IP: IgG) was performed as a control to exclude the non‐specific binding of antibodies to cellular proteins. Green arrows indicate non‐specific bands. The association between TLR3 and Trif, but not MyD88, was detectable in sham myocardium and was increased in infarct myocardium.

Secondly, to examine the endogenous TLR3 activity after MI, we determined the expression of TLR3 and its binding activity with Trif in infarct hearts. As shown in Fig. 3, both the mRNA and protein levels of TLR3 were significantly increased in the hearts of WT mice after 4 weeks of MI. Compared to the sham‐operated hearts, TLR3 mRNA levels were increased by 3.4‐ and 2.7‐folds (Fig. 3A), and TLR3 protein levels were increased by 5.9‐ and 4.4‐folds (Fig. 3B), respectively, in the infarct and remote zones of MI hearts. The immunohistochemical staining showed remarkably strong reactivity for TLR3 in cardiomyocytes of both the infarct and non‐infarct zones, while much less reactivity was shown for myocytes of sham‐operated hearts. Also, TLR3‐positive immunoreactivity was observed for part of the infiltrating cells in the infarct and border zones (Fig. 3C). Collectively, TLR3 expression in cardiomyocytes was enhanced after MI. In parallel to increased expression, the co‐IP assay revealed increased binding between TLR3 and Trif in infarct hearts (Fig. 3D), suggesting the activation of TLR3‐Trif signalling. Considering the pro‐autophagic effect of TLR3 in cultured cardiomyocytes (Fig. 1), the up‐regulated expression and signalling activity of TLR3 in MI indicate that TLR3 may potentially contribute to MI‐induced autophagy.

**Figure 4:**
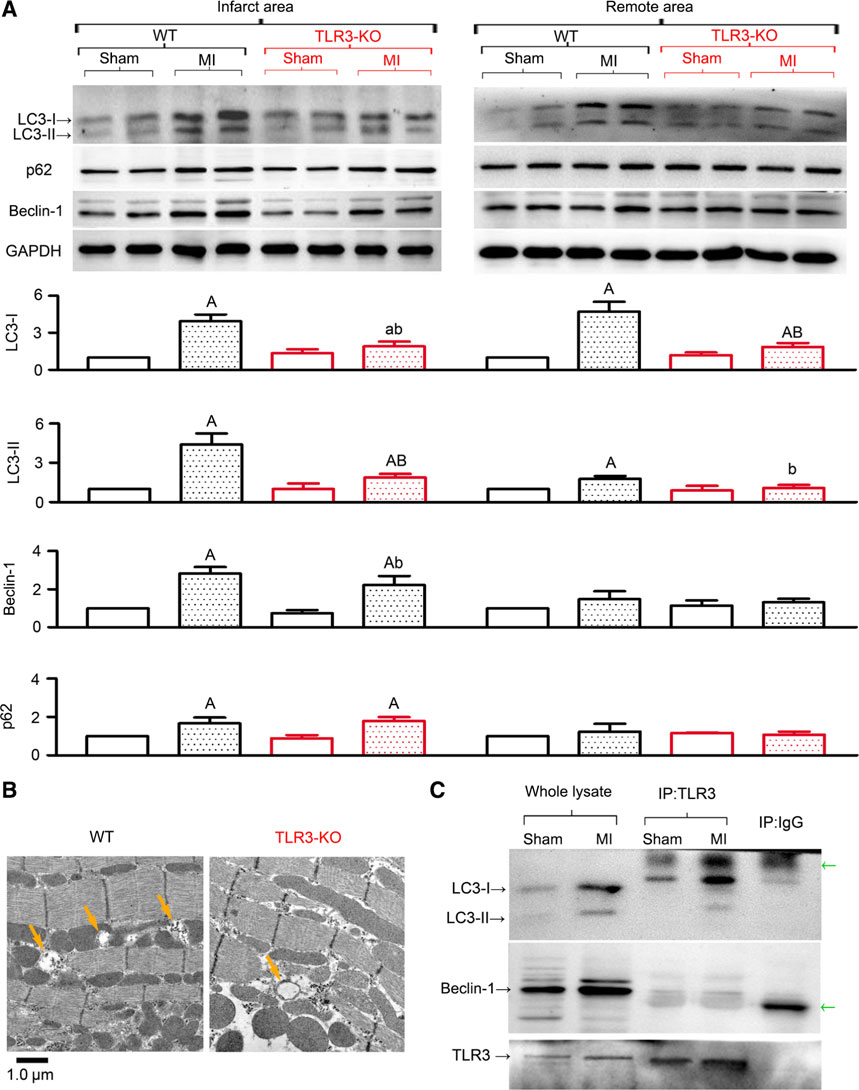
TLR3‐knockout (TLR3‐KO) attenuated cardiac autophagy induced by MI. The infarct and remote tissues were separately sampled from the left ventricle after 4 weeks of MI. Accordingly, anterior and posterior tissues of the left ventricle were sampled from sham hearts as controls. (**A**) Representative Western blot images and quantitative analyses of autophagy markers. *n *=* *4–8/group. Quantitative data are fold changes of WT‐sham. ^a^ *P* < 0.05, ^A^ *P* < 0.01 *versus* respective WT‐sham; ^b^ *P* < 0.05, ^B^ *P* < 0.01 *versus* respective WT‐MI. (**B**) Representative electron microphotographs of ultrathin sections of resin‐embedded heart biopsies. Arrows indicate autophagic vacuoles. (**C**) Lysates of infarct tissue were immunoprecipitated (IP), followed by SDS–PAGE and immunoblotting (IB) with indicated antibodies. IP with isotype IgG served as a control. Green arrows indicate non‐specific bands. Representative images were taken from four independent experiments.

Thirdly, to determine whether TLR3 contributes to MI‐induced autophagy, we examined cardiac autophagic activity under both normal and ischemic conditions in TLR3‐KO hearts. For sham‐operated hearts, no differences in autophagic markers were observed between WT and TLR3‐KO groups. In WT hearts subjected to 4 weeks of LAD ligation, significantly increased protein levels of LC3‐I, LC3‐II, beclin‐1 and p62/SQSTM1 were observed in the infarct area, and relatively small increases were observed in the remote area. TLR3‐KO hearts had uniformly decreased levels of LC3‐I, LC3‐II, beclin‐1 and p62/SQSTM1, typically in the infarct area (Fig. 4A). Also, morphological data obtained by electron microscopy showed that autophagic vacuoles, which were abundant in infarcted WT hearts (Fig. 4B), decreased in number by 67% in infarcted TLR3‐KO hearts (WT: 5.5 ± 1.9, TLR3‐KO: 1.8 ± 1.5 per 100 mm^2^, *P *<* *0.01). It is shown here that autophagic activity was decreased in TLR3‐KO hearts, suggesting that endogenous TLR3 promotes cardiac autophagy after MI.

Fourthly, we further examined the physical association between TLR3 and two proteins essential for autophagy initiation, LC3 and beclin‐1. The co‐IP assay (Fig. 4C) showed a detectable binding between TLR3 and LC3‐I in sham hearts of WT mice, which became more evident in infarct hearts. In contrast, no visible binding was observed between TLR3 and beclin‐1. These data support that TLR3 is involved in MI‐induced autophagy.

### TLR3‐knockout attenuated heart failure and improved survival in mice subjected to MI

To uncover the functional role of TLR3‐mediated autophagy in MI, we compared cardiac morphology, function and survival rate between WT and TLR3‐KO mice. The mice died within the observation period (4 weeks) were only counted for the calculation of survival rate, but exclusively excluded from the other analyses.

**Figure 5:**
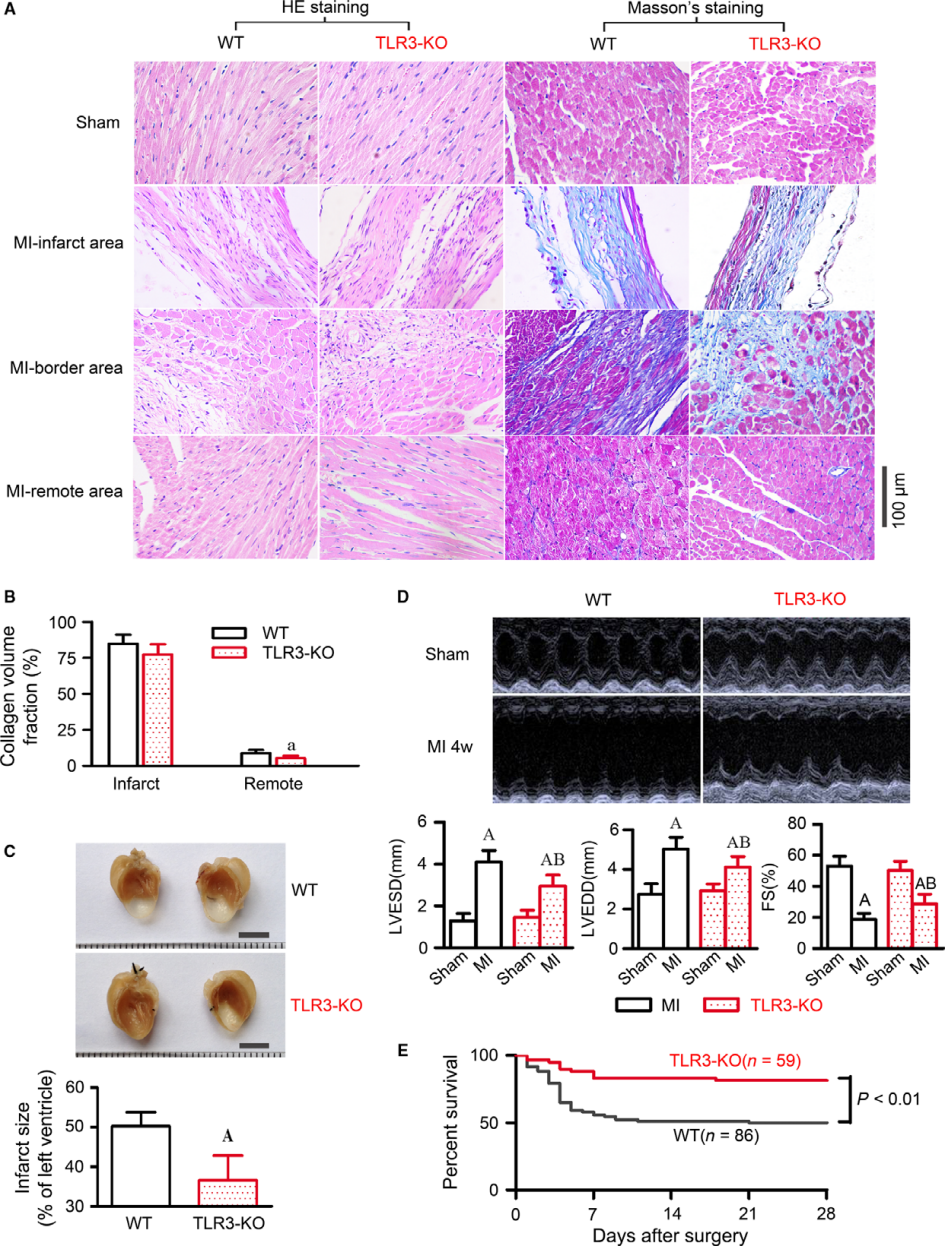
TLR3‐KO attenuated heart failure and improved survival in mice subjected to MI. All measurements were performed after 4 weeks of MI, and quantitative data are expressed as means ± S.D. (**A**) Representative microscopic images of haematoxylin and eosin (HE) and Masson's trichrome staining for heart sections. Images were taken from 3–6 mice/group. (**B**) Collagen volume fraction calculated from Masson's trichrome staining images, expressed as a percentage of the total left ventricular myocardial volume. *n *=* *6 mice/group. ^a^ *P* < 0.05 *versus* WT. (**C**) The upper panel shows representative gross view images of mouse heart coronally sectioned in halves, presenting the infarct area in whitish colour. The lower panel shows infarct size of post‐infarct hearts, determined with a length‐based approach as described in the methods. *n *=* *6 mice/group. ^A^ *P* < 0.01 *versus* WT. (**D**) Representative M‐mode ultrasound tracings of sham and MI hearts taken at the midpapillary level, and quantitative analysis of echocardiographic parameters including left ventricular end‐systolic diameter (LVESD), left ventricular end‐diastolic diameter (LVEDD) and fractional shortening (FS, %) of the left ventricle. *n *=* *13–15 mice/group. ^A^ *P* < 0.01 *versus* WT‐sham, ^B^ *P* < 0.01 *versus* WT‐MI. (**E**) Kaplan–Meier survival curves after coronary‐ligation surgery in WT and TLR3‐KO mice.

The histological staining for HE and Masson's trichrome revealed no morphological difference between WT and TLR3‐KO hearts receiving sham operation. However, the cardiac injury induced by MI was morphologically improved in the absence of TLR3. In Masson's staining, significant fibrosis manifested by large blue areas was observed for the infarct area, while mild fibrosis was observed for the remote area in both WT and TLR3‐KO hearts (Fig. 5A). The collagen volume fraction calculated from microscopic Masson's images was comparable in the infarct area between WT and TLR3‐KO groups, whereas smaller in the remote area of TLR3‐KO hearts than that of WT hearts (Fig. 5B). The infarct size of WT mice was 50.2 ± 3.6 %, which was significantly decreased to 36.6 ± 6.2 % in TLR3‐KO mice (Fig. 5C).

In the M‐mode ultrasound images taken at the midpapillary level, sham‐operated WT and TLR3‐KO mice showed similar parameters. After 4 weeks of MI, significant increases in left ventricular end‐systolic and end‐diastolic diameters, with a large decrease in fractional shortening, were seen for WT mice. In contrast, the above changes were consistently attenuated in TLR3‐KO mice (Fig. 5D). These data suggest that TLR3‐KO attenuated congestive heart failure derived from MI.

The survival rates after MI were compared up to 4 weeks between WT and TLR3‐KO mice. The rate of death during surgery was approximately 10%, with no difference between WT and TLR3‐KO mice. After the surgery, all the sham‐operated mice, either WT (*n *=* *20) or TLR3‐KO (*n *=* *18), survived through the observation period of 4 weeks. However, only 50% of the infarcted WT mice survived by 4 weeks. The knockout of TLR3 significantly increased survival rate to 81.4% (Fig. 5E).

### Autophagy induction abolished the protection of TLR3‐knockout against MI

**Figure 6:**
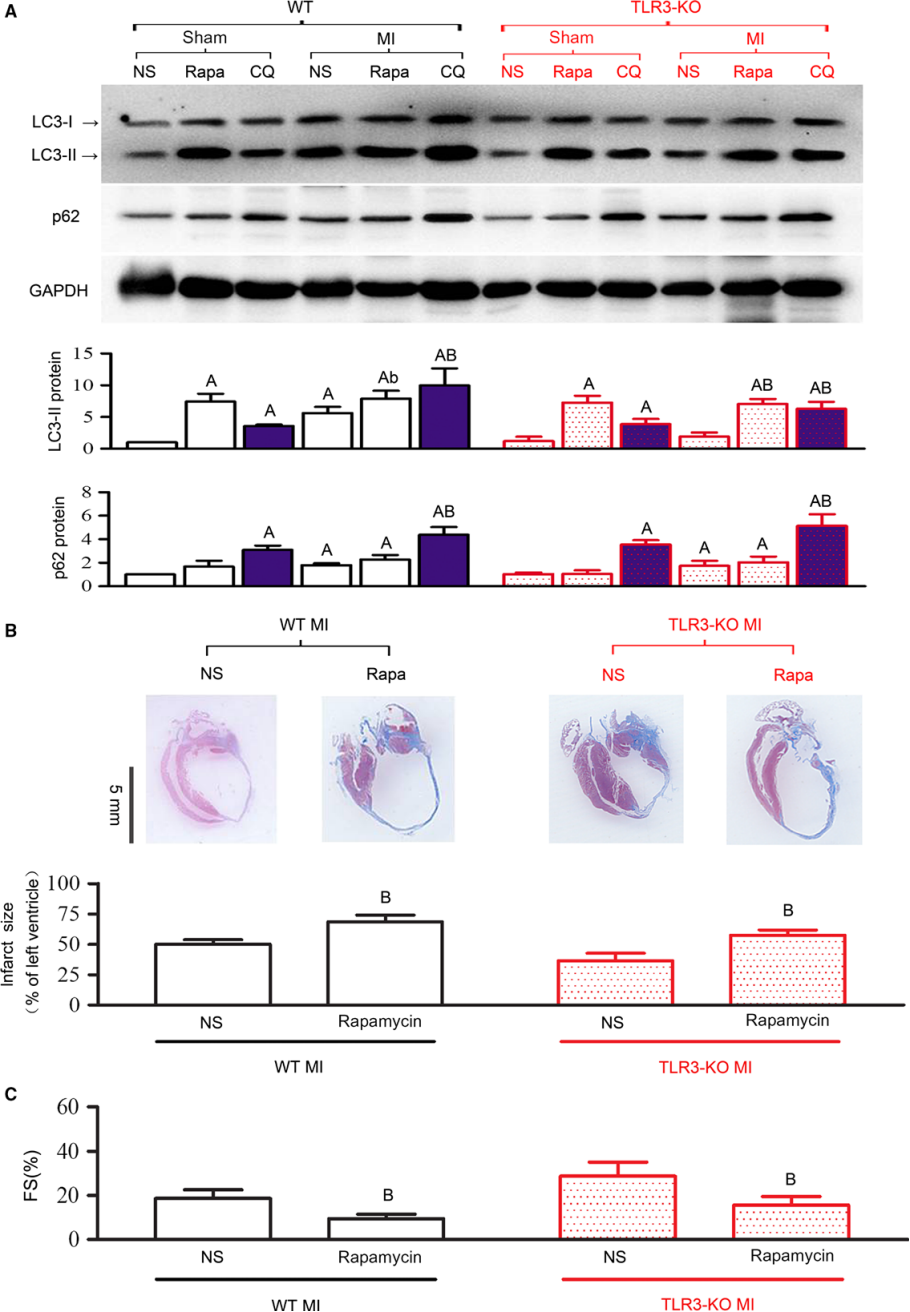
Autophagy induction abolished the protection of TLR3‐KO against MI. An autophagy inducer rapamycin (Rapa, 2 mg/kg/day) or an autophagic flux inhibitor chloroquine (CQ, 50 mg/kg/day) was daily intraperitoneally injected for 2 weeks, starting from day 1 after surgery. Normal saline (NS) was injected as control. Measurements were taken at 2 weeks. (**A**) Representative Western blot images and quantitative data of LC3 and p62 proteins in infarct tissue. Rapamycin increased LC3‐II in all the groups, suggesting successful induction of autophagy. CQ (blue bars) induced similar accumulations of LC3‐II and p62 in WT and TLR3‐KO myocardium, suggesting that autophagy flux was comparable between the two groups. (**B**) Representative coronal‐sectional images of Masson's trichrome staining and infarct size of post‐infarct hearts. (**C**) Fractional shortening (FS, %) of the left ventricle. All data are means ± S.D. *n *=* *4–6 mice/group. ^A^ *P* < 0.01 *versus* respective ‘sham+NS’; ^b^ *P* < 0.05, ^B^ *P* < 0.01 *versus* respective ‘MI+NS’.

To further examine whether reduced autophagy in TLR3‐KO mice contributes to improved survival and heart protection against MI, we applied an autophagy inducer rapamycin daily for 2 weeks, starting from 24 hrs after LAD ligation. The results showed that rapamycin effectively increased autophagic activity in both sham and LAD‐ligated mice (Fig. 6A). Coinciding with the induction of autophagy, the infarct size was enlarged (Fig. 6B), and the post‐infarct heart function was deteriorated in TLR3‐KO mice (Fig. 6C). These results demonstrate that inhibition of autophagy in the absence of TLR3 is protective against MI‐induced heart failure and lethality.

To verify whether TLR3 affect autophagic flux *in vivo*, we intraperitoneally injected CQ for 2 weeks, and observed similar accumulations of LC3‐II and p62 in WT and TLR3‐KO myocardium (Fig. 6A). This result, in accordance with that in cultured myocytes (Fig. 1C), suggests that autophagy flux is not affected by TLR3‐KO.

**Figure 7:**
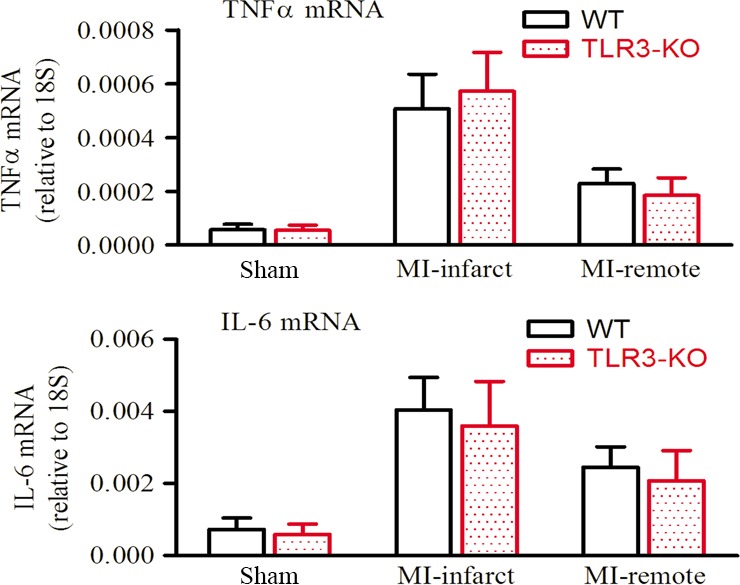
Cardiac cytokine expression in mice was unaffected by TLR3‐KO. The mRNA levels of inflammatory cytokine markers tumour necrosis factor α (TNFα) and interleukin‐6 (IL‐6) in heart tissue after 4 weeks of myocardial infarction were determined by real‐time RT‐PCR and normalized to 18S ribosome RNA transcript levels. Data are means ± S.D. *n *=* *4–5 mice/group.

TLR3 is known to regulate cellular inflammation *via* the transcription factors NF‐kB and IRF3 2, 26 and play an essential role in virus‐induced cardiac inflammation 27, 28. To discriminate the potential role of TLR3‐mediated inflammation in MI, we determined cardiac expression of inflammatory cytokines in the presence or absence of TLR3. As shown in Fig. 7, the basal level of cardiac cytokine expression is comparable between WT and TLR3‐KO mice, and MI induced similar increases in both groups. In line with this result, while robust inflammatory cell infiltration was present in the infarct and border areas of WT hearts, there was no visible difference in TLR3‐KO hearts, as shown by the HE and Masson's staining (Fig. 5A). These data indicate that myocardial inflammation is not affected by TLR3‐KO. Therefore, the involvement of inflammation in TLR3‐KO‐mediated autophagy inhibition and cardiac protection can be excluded.

## Discussion

TLRs are a family of innate immune receptors that are essential for recognizing PAMP and DAMP molecules. They are expressed by a variety of immune and non‐immune cells including cardiomyocytes 9. Although autophagy has been linked to TLR signalling, most of the knowledge was obtained from immune cells 5, 6, 7, 8. The action of TLRs on autophagy in cardiomyocytes remains unknown. This study treated cultured cardiomyocytes with a TLR3 agonist, either alone or in the presence of a lysosomal inhibitor, and observed that autophagy induction was stimulated by TLR3, whereas autophagic flux remained intact. Pathway dissection using siRNA knockdown techniques showed that TLR3 induced autophagy through the Trif‐dependent pathway, which was verified by the co‐IP analysis showing physical association between TLR3 and Trif but not MyD88. To identify the potential autophagy‐inductive role of TLR3 *in vivo*, the present study employed the mouse model of MI, a condition that has been described to induce autophagy 10, 25. We observed that over 4 weeks of MI, cardiac autophagy was persistently enhanced, accompanied with increased expression of TLR3 and its association with Trif. The knockout of TLR3 significantly attenuated autophagy, prevented heart failure and improved survival, which was abolished by an autophagy inducer. Taken together, this study shows that TLR3 plays a role in persistent autophagy after MI, which contributes to heart failure and lethality.

Autophagy is an essential process for cells to maintain homoeostasis. It enables cells to clean their interiors by forming double‐membraned organelles called autophagosomes, which deliver excessive or aberrant organelles and protein aggregates to the lysosomes for degradation 29. During cell starvation or stress, autophagy is required for organelle turnover, protein degradation and recycling of cytoplasmic components. As a common process, autophagy has been widely characterized in various cell types including cardiomyocytes 30. It is notable that proper autophagic activity is critical for normal maintenance of cardiac homeostasis. Either excessive or insufficient levels of autophagic flux can contribute to cardiac pathogenesis 10. A variety of cardiac stresses, including ischemia‐reperfusion, pressure overload and heart failure, have been shown to be related to autophagy. Evidence of autophagy in human heart diseases was first reported in patients with dilated cardiomyopathy 31, and later in patients with other cardiac disorders 32, 33, 34.

Autophagy is a highly dynamic process that needs to be carefully assessed. Commonly used measurements for autophagy include Western blot for LC3 and detection of autophagic puncta. The conversion of LC3‐I to LC3‐II through lipidation is a recognized hallmark of autophagy induction, which can be monitored by Western blot that identifies the electrophoretic mobility shift from the slower‐migrating non‐lipidated LC3‐I to faster‐moving lapidated LC3‐II 19, 20. The formation of autophagosomes is essential in autophagy detection, which can be measured by electron microscopy and puncta formation of fluorescent‐tagged LC3 proteins in cytoplasm under fluorescence microscopy. However, snapshot measurements of LC3 and autophagosomes without measuring autophagic flux are incomplete. A recent review by Gottlieb *et al*. elaborated on autophagy measurements and emphasized the need to assess autophagic flux, which can be assessed by a tandem RFP‐GFP‐LC3 construct and be inferred directly through lysosomal blockade or indirectly from the level of p62/SQSTM1 21, 35.

The activation of TLR3 was previously observed to induce autophagy in immune cells such as macrophages 8, 36, 37, while little is known in non‐immune cells. This study for the first time described a role of TLR3 in cardiac autophagy and examined the effect of TLR3 on autophagic flux. The induction of autophagy by TLR3 in immune cells was judged based on the evidence of LC3‐II formation and autophagosome accumulation 6, 7, 8. This study observed similar results in cardiomyocytes (Fig. 1). However, previous observations were limited to snapshot measurements of LC3 and autophagosomes. Given that enhanced autophagy initiation or decelerated autophagic flux each may cause increases in LC3‐II and autophagosomes 21, it is necessary to examine whether TLR3 affects autophagic flux. We herein addressed this question. Using a tandem mRFP‐GFP‐LC3 adenovirus, we detected no change in autophagic flux (Fig. 1B). Also, using CQ to block autophagic flux, we observed further increases of p62 in response to TLR3 activation (Fig. 1C), suggesting that autophagic flux remains intact upon TLR3 activation.

As to the signalling cascade leading to autophagy induction after TLR activation, previous studies in immune cells have shown the requirement for MyD88 and TRIF in immunological autophagy mediated by different TLR subtypes 7, 8, 36. Shi *et al*. showed that TLR3 signalling uses Trif, but not MyD88, to trigger autophagosome formation in macrophages 36. In accordance with this, we observed physical association between TLR3 and Trif, but not MyD88, in sham heart tissue, which was increased in infarct hearts (Fig. 3C). In cultured cardiomyocytes exposed to TLR3 agonists, the ablation of Trif, rather than MyD88, remarkably prevented autophagy induction (Fig. 1D). Collectively, it is indicated that cardiac TLR3 signalling is dependent on Trif rather than MyD88, and Trif links cardiac TLR3 to autophagy induction.

Multiple studies have demonstrated that cardiomyocyte autophagy is activated during myocardial ischaemia 10. However, whether the up‐regulated autophagy is adaptive or maladaptive is not well defined. While some studies show cardioprotective effects of autophagy under ischaemic stress 38, 39, 40, analysis of hearts from patients with end‐stage heart failure suggests autophagic death as the most prominent mechanism for the death of cardiomyocytes 41, 42. Minatoguchi's group observed persistent increases in LC3‐II and p62 in infarct hearts over the observation period of 3 weeks, and the most active formation of autophagosomes in remote areas at 3 weeks, as shown by LC3‐positive dots in immunofluorescence staining 25. The present study observed similar up‐regulation of LC3‐II and p62 following MI (Fig. 2), except that relatively high levels of LC3‐II and p62 were observed for infarct areas, compared to remote areas (Fig. 4A). This discrepancy might be the result of different assay methods. Besides, Minatoguchi's group reported that food restriction (FR) prevented post‐infarction heart failure by ‘enhancing autophagy’ 43, whereas we claim here that TLR3‐KO generated similar protection by ‘inhibiting autophagy’. These results appear paradoxical, but may be explained from different aspects of autophagy dynamics. In the FR study, increased ratio of LC3‐II/LC3‐I was used as an indicator of enhanced autophagic activity 43. Although this ratio has been used by many studies, it is actually fickle and unreliable, as pointed out by a recent review 21. Instead, the level of LC3‐II normalized to a protein loading control is proper for flux measurements. When just looking at LC3‐II shown by the Western blot images in the FR study (Fig. 3B in reference 43), we see that FR abolished the increase of LC3‐II induced by MI, as well as the increase of p62. Furthermore, LC3‐II and p62 in the FR group were both greatly increased after blockade with CQ. In our opinion, these data suggest that FR accelerates autophagic flux and reduced autophagic cargo accumulation, rather than ‘enhancing autophagy’. Differently, we herein observed that TLR3 stimulated autophagy induction without affecting autophagic flux. In addition, our results on rapamycin are in conflict with several previous reports 25, 44, 45. Kanamori *et al*., started daily injection of rapamycin after 2 weeks of MI, and detected protective effects after 1 more week 25. Buss *et al*. reported that everolimus, a drug similar to rapamycin, attenuated ventricular remodelling and dysfunction in rats subjected to MI 44. Wu *et al*. observed that rapamycin prevented MI‐induced NFκB activation and attenuated cardiac remodelling and dysfunction 45. We, on the contrary, observed damage of rapamycin. These discrepancies may result from differences in species, severity of MI and the timing and regimen of drug administration. More strikingly, the highly dynamic nature of autophagy may produce fickle results. Either over‐ or under‐activated autophagy and/or autophagic flux could be harmful. This likely renders great difficulties to intervene with autophagy‐associated diseases.

A remarkable effect downstream of TLR activation is the innate immune response, manifested as the production of inflammatory cytokines. TLR2 and TLR4 have been demonstrated to mediate cardiac inflammatory responses under ischaemic stress 9, 46. To examine whether TLR3 contributes to MI‐induced inflammation, we herein determined inflammatory cytokine expression in WT and TLR3‐KO hearts. The results showed comparable levels under both basal and MI conditions (Fig. 7). Inflammatory cell infiltration was also similar within WT and TLR3‐KO hearts subjected to MI (Fig. 5A). These data suggest that TLR3 is not involved in myocardial inflammation after MI. Previous studies testing TLR3‐KO mice in the model of myocardial ischemia/reperfusion (I/R) have reported conflict results. Chen *et al*. reported that TLR3‐Trif signalling had no impact on myocardial cytokines or neutrophil recruitment after I/R 47, but Lu *et al*. reported reductions of cytokine production as well as inflammatory cell infiltration in TLR3‐KO hearts subjected to I/R 48. We previously observed that TLR4 played a role in MI‐induced inflammation 49. However, the present study failed to detect a role for TLR3. An underlying reason might be that TLR3 is minor in cardiac inflammation, whereas TLR2 and TLR4 are predominant. Despite that, this study demonstrates the induction of cardiac autophagy upon TLR3 activation, which contributes to the persistently activated autophagy, heart failure and lethality following MI.

In summary, the present study first observed that TLR3 stimulated autophagy induction in cardiomyocytes, without affecting autophagic flux. In the mice subjected to MI‐induced persistent autophagy, TLR3‐KO attenuated autophagy, reduced infarct size and improved heart failure and survival. It is highlighted that immunoreceptors may play an important role in post‐MI heart failure and lethality through regulating autophagy, while the underlying molecular signals need more investigation.

## Conflicts of interest

The authors confirm that there are no conflicts of interest.
